# Image Segmentation Based on the Optimized K-Means Algorithm with the Improved Hybrid Grey Wolf Optimization: Application in Ore Particle Size Detection

**DOI:** 10.3390/s25092785

**Published:** 2025-04-28

**Authors:** Xinyi Chai, Zijun Wu, Wei Li, Haowei Fan, Xinyang Sun, Jing Xu

**Affiliations:** 1 School of Automation, Jiangsu University of Science and Technology, No. 666 Changxiang Road, Zhenjiang 212100, China; chai_xinyi2025@163.com; 2 School of Mechanical Engineering, Jiangsu University of Science and Technology, No. 666 Changxiang Road, Zhenjiang 212100, China; 222241802331@stu.just.edu.cn (Z.W.); 18667923619@163.com (W.L.); yomje658@163.com (H.F.); 3 School of Naval Architecture and Ocean Engineering, Jiangsu University of Science and Technology, No. 2 Mengxi Road, Zhenjiang 212114, China; 19707332869@163.com; 4 Marine Equipment and Technology Institute, Jiangsu University of Science and Technology, No. 2 Mengxi Road, Zhenjiang 212008, China

**Keywords:** image segmentation, ore particle size detection, improved GWO, K-means

## Abstract

**:** Image segmentation is an important part of ore particle size detection, and the quality of image segmentation directly affects the accuracy and reliability of particle size detection. Due to the poor quality and low efficiency of ore particle image segmentation in ore size detection, developing a fast and accurate algorithm for segmenting ore particle images remains a global challenge. However, the quality of image segmentation is closely related to calculating ore density, improving beneficiation efficiency, and evaluating crushing effectiveness. In this paper, a novel image segmentation algorithm is proposed, combining the K-means algorithm with a hybridized IGK-means. Firstly, the IGWO_SOA, by introducing a nonlinear convergence factor and incorporating the migration and spiral search mechanisms of SOA, is applied to overcome the weakness of being sensitive to initial centroids of the traditional K-means. IGWO_SOA is utilized to iteratively search for the optimal values of the initial cluster centers, which are then output as the results for subsequent clustering segmentation. An industrial experiment was conducted for multiple comparisons, which proved that the IGK-means has the characteristics of better image segmentation quality and being insensitive to illumination. The PSNR of the images segmented by IGK-means can reach up to 24.24 dB, and the FSIM can reach up to 0.2733, which proves the superiority and practicality of the algorithm in this paper.

## 1. Introduction

Ore size detection plays a critical role in mineral processing and mining operations. Accurate measurement of ore particle size directly impacts the efficiency of crushing, grinding, and beneficiation processes, ensuring optimal resource utilization and cost-effectiveness. Proper size detection aids in assessing ore density, improving separation precision, and maintaining consistent product quality. Moreover, it facilitates better control of equipment performance, reducing energy consumption and operational downtime. Many scholars have pointed out that reliable ore size detection is becoming increasingly important for achieving sustainable and economically viable mining methods [1]. However, due to the harsh working environment of ore size detection and the complex characteristics of the ore itself, detection methods have consistently been a technical bottleneck.

**Figure 1:**
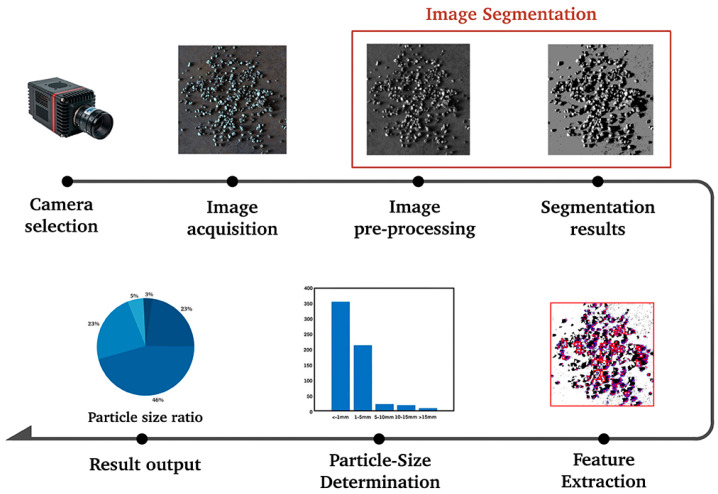
The process of particle size detection.

Since the introduction of ore particle size detection in the 1950s and 1970s, significant academic achievements have been made. Over the past few decades, more than a dozen detection methods have been proposed, including sieve analysis [2], laser diffraction [3], sedimentation analysis [4], and X-ray tomography [5], among others. These methods have greatly contributed to the theoretical and experimental development of particle size detection. However, they have also faced common challenges in practical applications. In [2], the labor intensity involved in handling large numbers of ore samples is very high, leading to inefficiencies and worker fatigue, which ultimately disrupts the continuity of the detection process. In [3,5], many of the instruments used are expensive and require stable environmental conditions to function properly. In certain mining sites, the presence of harsh environmental conditions—such as large temperature fluctuations, high humidity, and dust—can adversely affect the accuracy and reliability of the detection results. In [4], sedimentation analysis is time-consuming, and material characteristics such as the density and shape of the ore can influence the settling rate. In contrast, machine vision methods enable real-time acquisition of ore particle size information, providing immediate feedback and adjustments to the parameters of beneficiation equipment such as crushers. This not only improves beneficiation efficiency but also reduces energy consumption, making it widely adopted by researchers for particle size detection [6]. For machine vision-based ore particle size detection, first of all, a suitable camera should be selected according to the characteristics of the ore and the detection environment. Then, the lighting settings and image acquisition parameter settings are carried out. After that, image segmentation is performed through image preprocessing. Once the ore particles are segmented, their size features are extracted for particle size detection, The specific process of particle size detection is shown in Figure 1. Among the various techniques used in machine vision-based detection, image segmentation is one of the most essential and critical steps in image processing. Incomplete segmentation of ore particle images can severely interfere with the accuracy of the particle size distribution of ore particles. When an ore particle is segmented into multiple regions, these segmented parts may be misjudged as multiple independent ore particles. If only a part of an ore particle is segmented, it will lead to an underestimation of the actual particle size of the ore particle. Therefore, the quality and accuracy of ore particle image segmentation have a significant impact on particle size detection.

Nowadays, the main methods for ore particle image segmentation include thresholding [7,8], edge detection [9,10], region growing [11,12], the watershed algorithm [13,14], and deep learning techniques [15,16]. Thresholding is known for its good effectiveness, but its adaptability in varying lighting conditions and complex operational environments is relatively weak. Edge detection, on the other hand, is prone to generating false edges. The region growing algorithm, while simple in its segmentation principle, requires a significant amount of time to segment the entire image. The watershed algorithm is sensitive to weak edges, which helps preserve edge information, but it may lead to over-segmentation or under-segmentation in complex environments, thus reducing segmentation accuracy. Deep learning techniques have demonstrated remarkable advantages in the field of image segmentation. Among them, architectures such as U-Net have achieved high-precision segmentation across various domains, primarily due to their unique encoder–decoder structure and skip connection design. In [17], an improved model integrating the Swin Transformer into the U-Net architecture was proposed. By leveraging the global modeling capabilities of the Transformer, the model significantly improved the accuracy of semantic segmentation for remote sensing images. In [18], residual connections and attention mechanisms were introduced to form the RAR-U-Net model, which effectively addressed the challenge of spinal segmentation under noisy labels. This highlights the robustness of U-Net in scenarios with limited data quality. In [19], a U-Net-based deep learning model was successfully applied to the classification and segmentation of urban villages in high-resolution satellite images, further demonstrating the practical utility of this architecture in image segmentation tasks. In [20], an intelligent sorting scheme for coal and gangue that is adaptable to complex industrial scenarios was proposed by fusing multi-dimensional image features and combining machine learning or deep learning methods. This study is similar to the application concept of the aforementioned U-Net variants, both of which are committed to improving the segmentation accuracy through multi-modal feature fusion and model optimization. However, deep learning-based methods still face numerous challenges in practical applications. In the scenario of ore particle detection, a large amount of representative ore image data needs to be collected. Nevertheless, the complex and changeable environment of industrial sites makes it difficult to obtain high-quality and diverse data. Deep learning models have high requirements for hardware and need the support of high-performance GPUs, making it difficult to deploy them on industrial devices with limited resources. Existing models often have latency issues when processing high-resolution images or video streams in real time, failing to meet the stringent real-time requirements of scenarios such as industrial granularity detection.

Of the different methods, one of the most effective is the clustering method. The most widely applied clustering methods include fuzzy C-means [21], mountain clustering [22], K-means [23], and subtractive clustering [24]. The K-means algorithm is a kind of representative hard partitioning approach, which assigns data points to the nearest centroid. Then, positions of the centroids are iteratively updated based on their corresponding members to minimize the sum of squared errors. Many scholars have pointed out that K-means performs better than other clustering algorithms in practical applications [25]. In addition, K-means is widely applied medicine [26,27], agriculture [28,29], oceanography [30,31], and remote sensing [32,33]. However, the K-means algorithm has notable drawbacks, including high randomness and a tendency to get stuck in local optima, as well as the inability to effectively control the placement of cluster centers. In [34], in order to improve the calculation efficiency of K-means, a novel weighted bilateral k-means (WBKM) algorithm was proposed to cluster big data. On the basis of WBKM, a fast multi-view clustering algorithm with spectral embedding (FMCSE) was designed in [35]. To avoid falling into locally optimal clustering centers, many intelligent iterative optimization algorithms were applied. In [36], an improved PSO-K algorithm was applied for image segmentation, and the results of the experiments showed that it is possible to accurately segment the target from various kinds of agricultural images after the gray processing, which involves RGB with super green features. In [37], a hybrid algorithm combining the firefly algorithm and K-means (KM-FA) is proposed, with the Otsu criterion employed as the fitness function, demonstrating excellent performance in medical image applications. In [38], an improved colony optimization K-means (IABCKMC) was applied for selecting typical wind power output scenarios. It can be summarized from the literature that improvements in K-means mainly focus on reducing the calculation time and avoiding falling into local optimal clustering centers.

The grey wolf optimization (GWO) algorithm is a heuristic optimization method inspired by the social behavior model of grey wolves [39]. It incorporates adaptively adjustable convergence factors and an information feedback mechanism, enabling a balance between local exploitation and global exploration. Consequently, the GWO algorithm demonstrates strong performance in terms of solution accuracy and convergence speed. However, like other optimization algorithms, its convergence speed tends to decrease during the later stages of optimization. As iterations progress, other wolves (β, δ, and ω) continue to converge towards the leading wolves (α, β, and δ), which increases the likelihood of the algorithm becoming trapped in local optima [40]. In [41], in order to avoid the local optimal solution, a method that introduces a differential perturbation operator and improves the value assignment of control parameters is proposed to solve the problem of data clustering. In [42], An adaptive position update and circular population initialization (SGWO) method is applied to the parameter optimization of the Elman neural network, endowing the prediction method based on SGWO–Elman with accurate prediction performance and enabling it to excel in relevant data prediction tasks. In [43], an improved GWO algorithm with variable weights (VW–GWO) was proposed, and a new control parameter dominance equation was developed to reduce the likelihood of entrapment in local optima effectively.

Based on the above research, this paper aims to overcome the disadvantages of poor quality, low efficiency, and low reliability of previous methods by proposing a convenient and reliable segmentation method for ore particle images. Since the basic K-means clustering algorithm is prone to falling into local optima and is sensitive to the initial cluster centers, a hybrid method is adopted to optimize it. Moreover, this comprehensive optimization method combines the improvement of the convergence factor in the improved grey wolf optimizer with the seagull optimization algorithm (IGWO_SOA) to balance the search speed and population density. Specifically, in the process of updating the positions of the original grey wolf algorithm, some wolves are updated by introducing the migration mechanism and the spiral attack mechanism.

The rest of this paper is organized as follows. In Section 2, the basic theories of K-means, GWO, and SOA are presented. In Section 3, the iteration process of the proposed IGWO_SOA is described, and the segmentation method based on K-means and IGWO_SOA (IGK-means) is elaborated on in detail. In order to verify the accuracy and superiority of the improved algorithm in segmenting ore particle images, experimental analysis is presented in Section 4. Then, industrial experiments on ore particle size detection are also carried out in Section 4. Some conclusions and prospects are summarized in Section 5.

## 2. Background

### 2.1. K-Means Algorithm

The core idea of the K-means algorithm [44] is to divide a given dataset into K clusters according to similarity, ensuring that the data points within the same cluster are as similar as possible, while those in different clusters are as different as possible. The optimization objective of the K-means algorithm is to minimize the within-cluster sum of squares (SSE).

The K-means algorithm is an unsupervised classification algorithm. Suppose there is an unlabeled dataset:

(1) $$P=\left[p_{1},p_{2},\ldots ,p_{n}\right].$$

The task of this algorithm is to cluster the dataset into k clusters $C=C_{1},C_{2},\ldots ,C_{k},$ The minimum loss function is:

(2) $$E=\overset{k}{\underset{i=1}{\sum }}\underset{p\in C_{i}}{\sum }|\left|p-u_{i}\right|{|}^{2}.$$

The smaller the value of P, the higher the similarity of the data within the cluster and the better the clustering effect. Among them, $u_{i}$ is the center point of the cluster:

(3) $$u_{i}=\frac{1}{\left|C_{i}\right|}\underset{p\in C_{i}}{\sum }p.$$

The formula for updating the clustering center is shown as follows:

(4) $$C_{i}=\frac{i}{n}\overset{n}{\underset{j=1}{\sum }}P_{j}.$$

**Figure 2:**
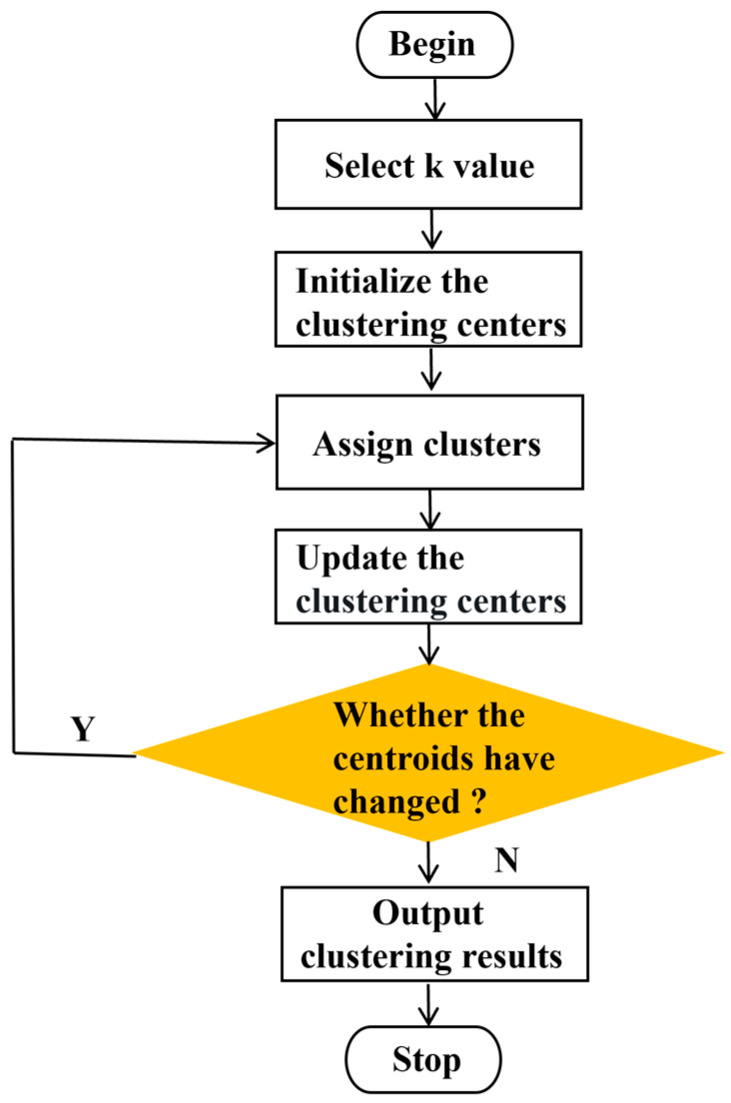
The process of the K-means algorithm.

The iterative process of the K-means algorithm can be generalized in Figure 2 and summarized as follows:

Step 1.1: Initialization. Initialize the number of clusters k and select data points from the dataset randomly as the initial clustering centers. Denote these initial centers as $C_{1},C_{2},\ldots ,C_{k}.$

Step 1.2: Assignment. Calculate the Euclidean distance between each point and the clustering center, and classify the nearest clustering center according to the minimum distance principle.

Step 1.3: Update. Calculate the current mean of each class to redetermine the center of clustering.

Step 1.4: Convergence Check. Calculate the difference between new and previous clustering centers. Stop the algorithm if the maximum difference is less than the stopping criterion or the max iterations are reached, taking the current cluster assignment and centers as results; otherwise, repeat steps 1.2, 1.3, and 1.4.

The flowchart of the K-means algorithm is shown.

### 2.2. Grey Wolf Optimizer Algorithm

The grey wolf optimizer (GWO) [45] is an optimization algorithm inspired by the behavior of grey wolves in nature. It was proposed by Seyedali Mirjalili and others in 2014. This algorithm searches for the optimal solution to optimization problems by simulating the social hierarchy structure and hunting strategies of grey wolves.

**Figure 3:**
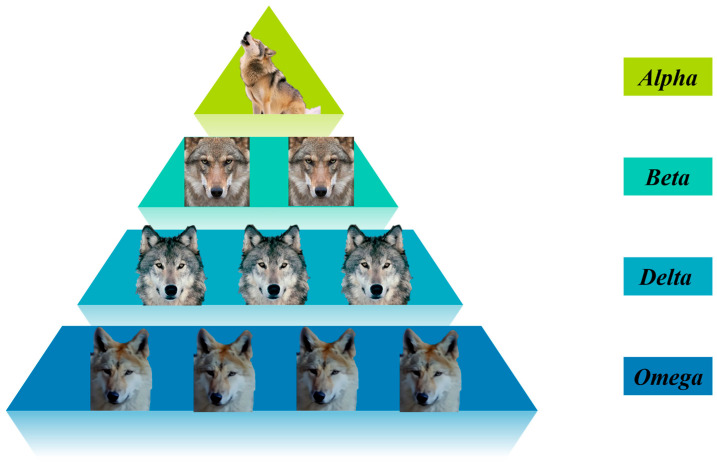
Grey wolf hierarchical structure.

The grey wolf population has a strict hierarchical system. As shown in Figure 3, wolves of different ranks account for different numbers in the wolf pack. There is usually only one alpha wolf, which serves as the core leader of the wolf pack; the number of beta wolves is relatively small, generally just a few, and they assist the alpha wolf; the number of delta wolves is relatively larger; and the omega wolves are the most numerous and form the main part of the wolf pack. The alpha wolf at the top of the pyramid represents the optimal solution in the current solution space. It is the leader of the group, responsible for making decisions and guiding the actions of the group. The beta wolves in the second layer of the pyramid are in the second-best position in the wolf pack. They assist the alpha wolf in hunting and leading the other grey wolves. When the alpha wolf is absent from the whole wolf pack, the beta wolf is the successor of the alpha wolf. The delta wolves in the third layer follow the instructions of the alpha wolf and the beta wolf to execute specific tasks, such as keeping watch and taking care of the cubs. In the algorithm, they represent the third-best solution. The old (less fit) alpha wolves and beta wolves are also demoted to the level of delta wolves. The omega wolves at the bottom are at the lowest level of the social hierarchy. They usually undertake the lowest-level jobs in the group, such as cleaning and being the last to receive food distribution. In the algorithm, they follow the guidance of the alpha wolf, the beta wolf, and the delta wolf to explore the search space. The mathematical expressions for the positions of the wolf pack during the hunting process are as follows:

(5) $$\begin{array}{c} D=|\vec{C}\cdot X_{p}\left(t\right)-X\left(t\right)|\\ X\left(t+1\right)=X_{p}\left(t\right)-\vec{A}\cdot D \end{array}.$$

where *t* is the current iteration number, $\vec{A}$ and $\vec{C}$ are coefficient vectors, and $X_{p}\left(t\right)$ and $X\left(t\right)$ are the position vectors of the prey and the grey wolf, respectively.

The specific process of GWO is as follows:

Step 2.1: Initialization. Randomly initialize the positions of N grey wolves within the solution space, considering the problem’s dimensionality D, and set the maximum number of iterations $\;t_{max}$.

Step 2.2: Fitness evaluation. Identify the top three candidates based on their fitness, assigning them the roles of α, β, and δ.

Step 2.3: Coefficient calculation. The coefficients $\vec{A}\;$ and $\vec{C}$ control the exploration and exploitation of the search process, the formulas for which are as follows:

(6) $$\vec{A}=2\vec{a}\cdot {\vec{r}}_{1}-\vec{a}.$$

(7) $$\vec{C}=2\cdot {\vec{r}}_{2}.$$

(8) $$a=2-\frac{2t}{t_{max}}.$$

where $t_{max}$ is the maximum number of iterations, and ${\vec{r}}_{2}\;$ is a random vector in the range [0, 1], controlling the balance of movement.

**Figure 4:**
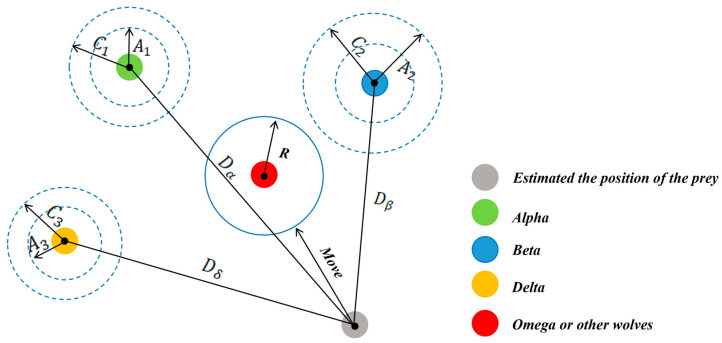
The position update of the grey wolves.

Step 2.4: Update position. Grey wolves update their positions based on the three best solutions and their distances from these leaders. The position update process is shown in Figure 4, and the process involves the following steps:

Calculate the distance vectors:

(9) $$D_{\alpha }=\left|C_{1}\cdot X_{\alpha }\left(t\right)-X\left(t\right)\right|.$$

(10) $$D_{\beta }=\left|C_{2}\cdot X_{\beta }\left(t\right)-X\left(t\right)\right|.$$

(11) $$D_{\delta }=\left|C_{3}\cdot X_{\delta }\left(t\right)-X\left(t\right)\right|.$$

where $X_{\alpha }\left(t\right)$, $X_{\beta }\left(t\right)$, $X_{\delta }\left(t\right)$ are the positions of the best, second-best, and third-best solutions, respectively.

Update intermediate positions:

(12) $${\vec{X}}_{1}={\vec{X}}_{\alpha }-{\vec{A}}_{1}\cdot \left({\vec{D}}_{\alpha }\right).$$

(13) $${\vec{X}}_{2}={\vec{X}}_{\beta }-{\vec{A}}_{2}\cdot \left({\vec{D}}_{\beta }\right).$$

(14) $${\vec{\;X}}_{3}={\vec{X}}_{\delta }-{\vec{A}}_{3}\cdot \left({\vec{D}}_{\delta }\right).$$

The new position of a wolf is calculated as:

(15) $$\vec{X}\left(t+1\right)=\frac{{\vec{X}}_{1}+{\vec{X}}_{2}+{\vec{X}}_{3}}{3}.$$

Step 2.5: Optimization iteration. If the wolves find the optimal value or the iteration number reaches the maximal *N*, the iteration process stops. Otherwise, steps 2.2 to 2.4 are repeated.

### 2.3. Seagull Optimization Algorithm

The seagull optimization algorithm (SOA) conducts evolutionary computations by imitating the behavioral habits of seagulls as living organisms [46]. The seagull individuals in the seagull algorithm mainly possess two mechanisms: migration and spiral attack. The migration operation involves displacing from the existing position to avoid collisions with other individuals. After moving to a new position, the seagull will approach the optimal position. The attack operation means that during the process of preying on the target, the seagull continuously changes its angle and attack speed, performing a spiral-shaped movement to update its position.

The seagull optimization algorithm is capable of adaptively adjusting its search strategy. It can make dynamic adjustments according to the current environment and search status, which enhances the flexibility of the algorithm. Moreover, it has a relatively strong global search ability and can effectively avoid getting trapped in local optimal solutions.

The mathematical formula for calculating the new position that does not collide with adjacent seagulls in the migration strategy is as follows:

(16) $$\vec{C_{s}}=K\times \vec{P_{s}}\left(x\right).$$

where $\vec{P_{s}}\left(x\right)$ represents the current position and K represents the movement behavior of seagulls in the search space.

When seagulls attack their prey, they perform spiral movements in the air, which can be abstracted into three-dimensional space.

(17) $$\left\{\begin{array}{l} x=rcos\left(\theta \right)\\ y=rsin\left(\theta \right)\\ z=r\theta \;\;\;\;\;\;\;\;\;\;\\ r=u\cdot e^{\theta v} \end{array}\right..$$

where u and *v* are hyperparameters and usually take the value of 1, r represents the spiral radius, and θ is a random angle value within the range of [0, 2π].

The new position of seagulls after attacking their prey is shown as follows:

(18) $$P_{s}\left(t\right)=D_{s}\left(t\right)\times x\times y\times z+P_{best}\left(t\right).$$

where $P_{best}\left(t\right)$ represents the current best position and $D_{s}\left(t\right)$ represents the distance between the search agent and the best search agent.

## 3. The Proposed Method

### 3.1. Improved Convergence Factor

When |$\vec{A}$| > 1, the grey wolf group will expand the search range to look for prey, that is, it will conduct global search with a fast convergence speed. When |$\vec{A}$| < 1, the grey wolf group will narrow the search range to attack the prey, that is, it will perform a local search with a slow convergence speed. Therefore, the magnitude of $\vec{A}$ determines the global search and local search capabilities of the GWO. It can be seen from Formula (5) that $\vec{A}$ changes with the change in the convergence factor a. The convergence factor a linearly decreases from 2 to 0 with the increase in the number of iterations. However, the process of the algorithm’s continuous convergence is not linear. From this, it can be understood that the linearly decreasing convergence factor a cannot fully reflect the actual optimization search process.

To address the above issues, this paper proposes an improved nonlinear convergence factor a, whose expression is shown in Equation (19) as follows:

(19) $$a=2\cos\left(\frac{\pi }{2}{\left(\frac{t}{t_{max}}\right)}^{\lambda }\right).$$

where λ is experimentally determined, t is the current iteration, and $t_{max}$ represents the maximum number of iterations.

**Figure 5:**
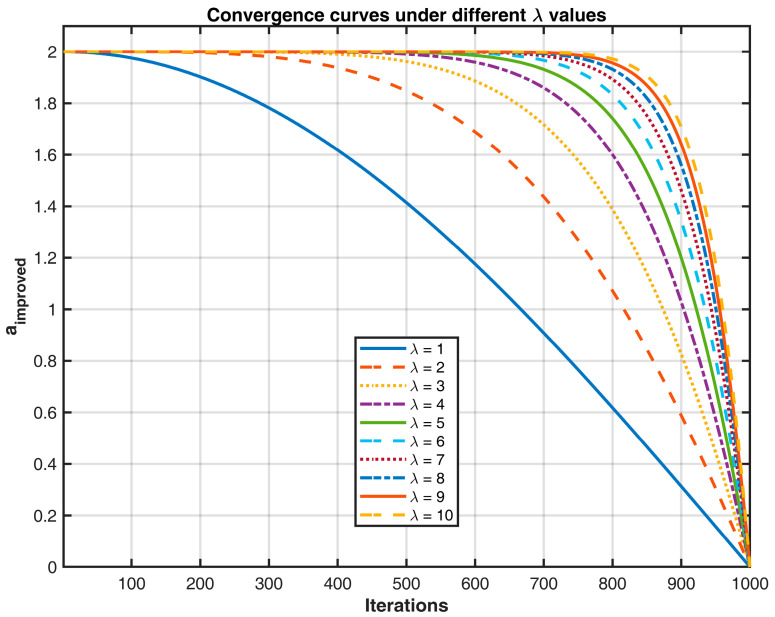
Convergence factor change curve.

When $t_{max}$=1000, different values of the parameter aa in Equation (19) were tested. The results for a are shown in Figure 5, where the curves from top to bottom correspond to λ = 1, 2, …, 10.

### 3.2. Hybrid Optimization Algorithm

In the later stage of optimization using the GWO, the optimization range of individual grey wolves becomes smaller as the wolf pack gradually surrounds the prey, a process during which the positions of individuals may collide and overlap. This reduction in position diversity, a factor contributing to the algorithm getting trapped in local optimal values, can be addressed by integrating mechanisms from the SOA. The migration behavior, characterized by its ability to prevent seagulls from gathering in positions, is employed to increase diversity and improve the algorithm’s capability to escape local optima. Additionally, the spiral search, noted for its fast speed and high efficiency, is incorporated. As a result, the migration mechanism and spiral search are combined to refine the experienced delta wolves in the wolf pack, thereby enhancing the algorithm’s optimization ability.

After fusing with the SOA, the position update formula for the delta wolf is shown as follows:

(20) $$\;\vec{X_{3}}=\left\{\;\begin{array}{c} \vec{X_{\delta }}+rd\vec{D_{\delta }}\;\;0\leq rd<0.5\\ \vec{X_{\delta }}+r\vec{D_{\delta }}cos\theta +r\left|\vec{X_{\delta }}-\vec{X_{t}}\right|sin\theta \;\;\;\;0.5\leq rd\leq 1 \end{array}\right..$$

where r is the helix radius, and rd is a random normal distribution factor within the interval [0, 1]: when 0≤rd<0.5, the migration mechanism is adopted to increase the randomness of the position update of the delta wolf, and when 0.5≤rd≤1, the spiral search mechanism is adopted. The position of the delta wolf is not only updated through the standard GWO but is also affected by the spiral radius, which can help the algorithm explore more effectively in the search space and thus find better solutions. The expression of θ in the formula is:

(21)  θ=rd⋅2π.

The implementation process of the improved GWO (IGWO_SOA) is as follows:

Step 3.1: Initialization. Set the algorithm parameters, providing the population size N, the maximum number of iterations $\;t_{max}$, and the radius of the spiral search r, etc.

Step 3.2: Fitness evaluation. Calculate the fitness values of all grey wolf individuals in the population. Select the top three wolves with the highest fitness values as the alpha wolf, beta wolf, and delta wolf, and record their positions $X_{\alpha }\left(t\right)$, $X_{\beta }\left(t\right)$, and $X_{\delta }\left(t\right)$, respectively.

Step 3.3: Update position. Update the positions of other grey wolf individuals in the population according to Formulas (12), (13), (15), and (20). Update $\vec{A}$ and $\vec{C}$ according to Formulas (6) and (7).

Step 3.4: Optimization iteration. If the wolves find the optimal value or the iteration number reaches the maximal N, the iteration process stops and the global optimal solution is output; otherwise, steps 3.2 to 3.4 are repeated.

**Figure 6:**
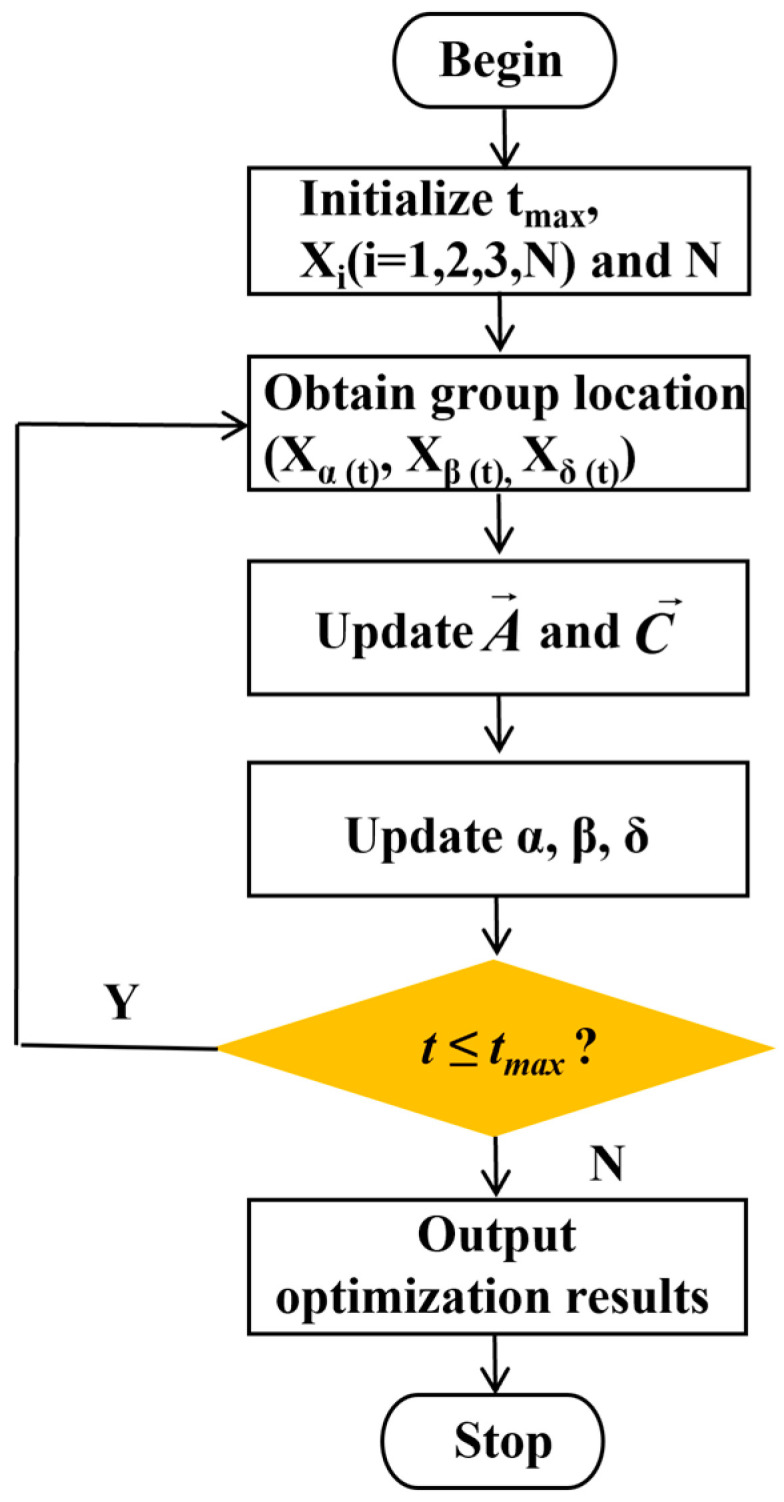
Iterative process of the proposed IGWO_SOA.

The flowchart of the IGWO_SOA is presented in Figure 6. For a comprehensive assessment of the performance of IGWO_SOA, kindly refer to Appendix A. Meanwhile, the in-depth discussion on the adaptability of λ can be found in Appendix B.

### 3.3. Image Segmentation Based on IGK-Means

When using the improved grey wolf algorithm to improve the K-means algorithm for image segmentation, the clustering centers are taken as the optimization variables of the grey wolf algorithm. Firstly, the target image is regarded as an n-dimensional spatial vector with the data sample point set  P, and *m* data points are randomly selected from it as the initial clustering centers. Then, the remaining data points in the set P are assigned to these m classes. Let $P_{j}$ be the *j*-th data point in the set P, $C_{i}$ be the *i*-th clustering center, and assume that $∥M_{i}-C_{j}∥$ is the smallest at this time. Then, the data point $P_{i}$ is assigned to the j-th class. The design of the fitness function is as follows:

(22) $$\;fitness=\overset{n}{\underset{i=1}{\sum }}\overset{m}{\underset{j=1}{\sum }}|\left|P_{j}-C_{i}\right|{|}^{2}.$$

After IGWO_SOA reaches the maximum number of iterations, the global optimal value obtained through optimization is used to initialize the clustering centers of the next process, the K-means clustering algorithm, thus overcoming the disadvantages of the K-means clustering algorithm, such as being sensitive to the initialization of clustering centers.

The image segmentation based on the combination of the IGWO_SOA-optimized K-means algorithm (IGK-means) mainly consists of two steps: Optimize the initial clustering centers through the global search ability of IGWO_SOA to find the reasonable and optimal initial clustering center positions in the image point set. Then, utilize the K-means clustering for local optimization until the end of the iteration. The implementation steps of the algorithm are as follows:

Step 4.1: Initialization. Set the number of populations N and clusters k, the size of the spiral search *r*, and the maximum number of iterations $\;t_{max}$. Randomly initialize the positions of N grey wolves within the solution space.

Step 4.2: Fitness evaluation. According to the fitness function $\overset{n}{\underset{i=1}{\sum }}\overset{m}{\underset{j=1}{\sum }}|\left|P_{j}-C_{i}\right|{|}^{2}$, wolves are ranked based on their fitness values. The top three wolves are designated as α, β, and δ, representing the best, second-best, and third-best solutions, respectively. The remaining wolves, known as ω, follow the guidance of α, β, and δ in the optimization process.

Step 4.3: Obtain group location. Wolves mathematically encircle their prey by adjusting their positions based on the prey’s location, using coefficient vectors $\vec{A}$ and $\vec{C}$.

Step 4.4: Update position. During the hunting phase, wolves update their positions, and then the results are averaged.

Step 4.5: Iterative optimization. The iteration process terminates either when the wolves identify the optimal value or when the maximum number of iterations N is reached. If neither condition is met, steps 4.2 to 4.4 are repeated until convergence.

Step 4.5: Obtain the initial clustering center. Output the optimal value obtained from the IGWO_SOA algorithm iteration as the initial cluster centers for the K-means algorithm.

Step 4.6: Assignment. Compute the Euclidean distance between each point and the cluster centers, then assign each point to the nearest cluster center based on the minimum distance principle.

Step 4.7: Update. Calculate the mean of the points in each cluster to update and redefine the cluster centers.

Step 4.8: Convergence check. Compute the difference between the new and previous cluster centers. If the maximum difference is smaller than the stopping threshold or the maximum number of iterations is reached, the algorithm terminates, and the current cluster assignments and centers are taken as the final results. Otherwise, repeat steps 4.6 to 4.8.

**Figure 7:**
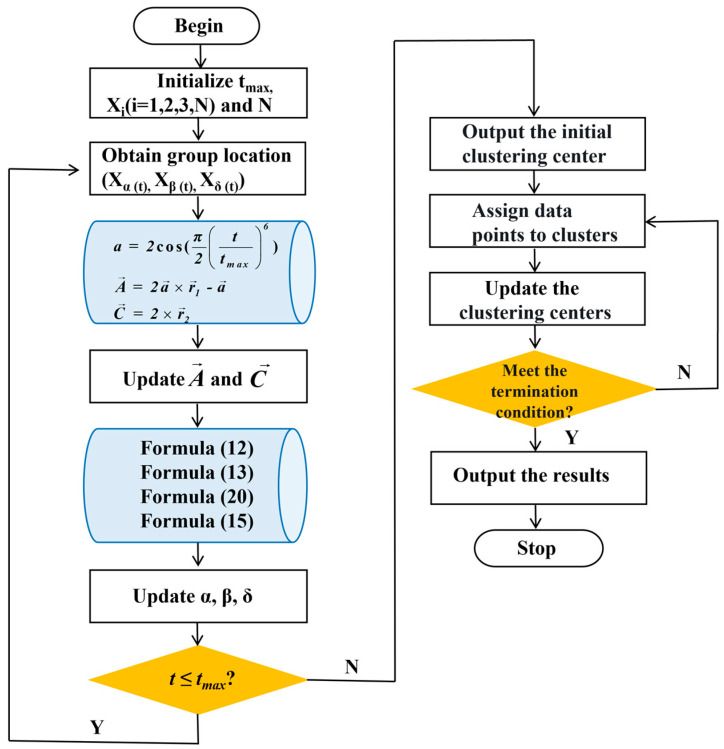
Flowchart of the IGK-means.

The flowchart of the IGK-means is shown in Figure 7.

## 4. Experiment and Analysis

### 4.1. Experimental Platform Construction

**Figure 8:**
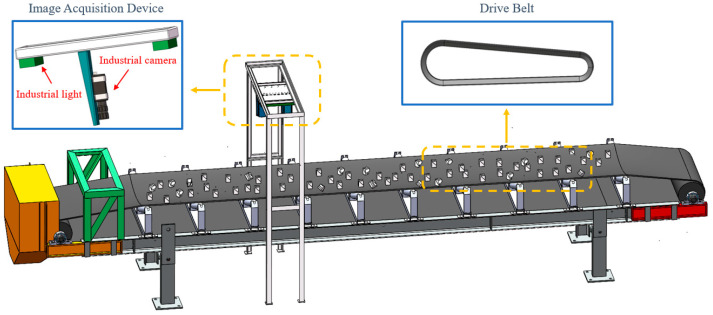
Ore particle size detection system.

**Figure 9:**
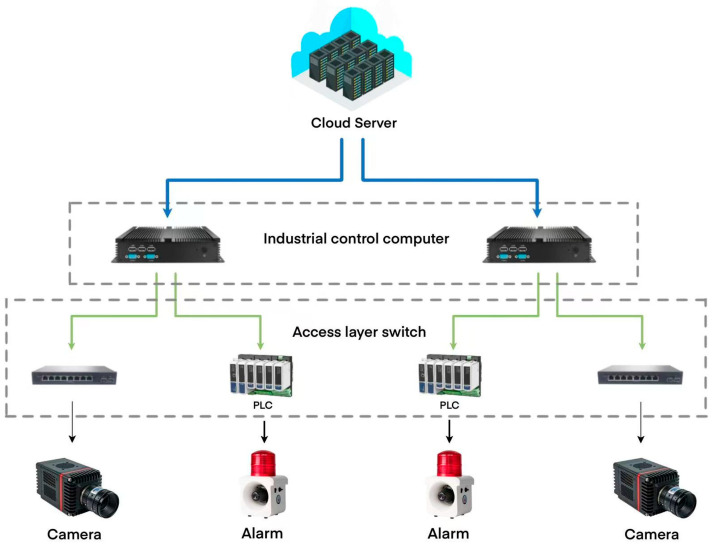
The network cable structure of the ore particle size detection system.

**Figure 10:**
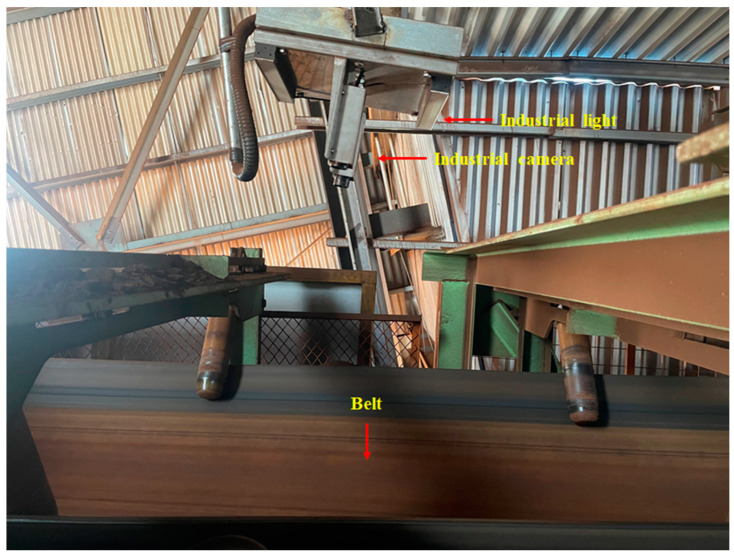
The experimental site.

In order to validate the superiority and effectiveness of the proposed method, an experimental platform for ore particle collection was constructed, located in the Marine Equipment Research Institute of Jiangsu University of Science and Technology, Zhenjiang City, Jiangsu Province, China, as shown in Figure 8. The platform primarily consists of industrial cameras, industrial light sources, and a track system. The network structure of this system is shown in Figure 9. It is important to ensure good ventilation around the camera during operation to prevent prolonged high-temperature conditions. Figure 10 shows the actual working scene of the system.

During the experiment, sample images of ore particles were captured using an industrial camera. Subsequently, the sample images were segmented using the method proposed in this paper. To comprehensively evaluate the performance of the proposed method, K-means, GK-means, KM-FA, and IGK-means were introduced for comparative analysis.

Segmentation experiments were conducted on 400 ore particle images collected from the conveyor belt using the proposed IGK-means method. For comparison, segmentation experiments were also performed using the K-means algorithm, mountain clustering (MC) the GWO-optimized K-means algorithm (hereafter referred to as GK-means), and the KM-FA algorithm described in [37], to validate the advantages of the proposed method in ore particle image segmentation.

This study employs two widely used evaluation metrics to assess the performance of image segmentation: peak signal-to-noise ratio (PSNR), feature similarity index measure (FSIM), intersection over union (IoU), and pixel accuracy (PA). PSNR is a direct metric for measuring the quality of reconstructed images, providing a quantitative evaluation of the fidelity of segmented images compared to the original. The FSIM evaluates the structural similarity between images, emphasizing the preservation of essential image features, thereby offering a comprehensive assessment of the segmentation quality. IoU is a commonly used evaluation metric in object detection and image segmentation tasks, which is used to measure the overlapping degree between the prediction result and the ground truth label. PA reflects the proportion of the number of pixels correctly predicted by the model in the total number of pixels.

(23) $$\;\mathrm{PSNR}=10\cdot \log_{10}\left(\frac{{\mathrm{MAX}}^{2}}{\mathrm{MSE}}\right).$$

(24) $$\mathrm{MSE}=\frac{1}{MN}\overset{M}{\underset{i=1}{\sum }}\overset{N}{\underset{j=1}{\sum }}{\left[I\left(i,j\right)-I_{\mathrm{ref}}\left(i,j\right)\right]}^{2}.$$

where MAX represents the maximum possible pixel value of the image, such as 255 for an 8-bit grayscale image. *M* and *N* denote the dimensions of the image, while $I\left(i,j\right)$ represents the pixel value at the $\left(i,j\right)$ position in the segmented image and $I_{\mathrm{ref}}\left(i,j\right)$ denotes the corresponding pixel value in the reference image.

(25) $$\mathrm{FSIM}=\frac{\sum_{x\in \Omega }S_{L}\left(x\right)\cdot PC_{m}\left(x\right)}{\sum_{x\in \Omega }PC_{m}\left(x\right)}.$$

where $S_{L}\left(x\right)$ represents the similarity between the luminance features of the segmented image and the reference image, while $PC_{m}\left(x\right)$ denotes the phase congruency at pixel x, capturing the structural information of the image. Ω refers to the set of all pixels in the image.

(26) $$IoU=\frac{\left|m\cap n\right|}{\left|m\cup n\right|}.$$

where *m* represents the predicted region, and *n* represents the ground truth region.

(27) $$PA=\frac{\sum_{i=0}^{n}q_{ii}}{\sum_{i=0}^{n}\sum_{j=0}^{n}q_{ij}}.$$

where *n* represents the number of classes in the image, and $q_{ii}$ represents the number of pixels that are correctly classified into the *i*-th class.

In order to compare the impact of different algorithms for image segmentation on particle size detection, taking the results of manual screening as the reference standard, the deviation rate is adopted to evaluate the accuracy of particle size detection. The deviation value is calculated based on the number of ore particles.

(28) $$\varepsilon =\frac{\left|H-E\right|}{E}\times 100\%.$$

where ε represents the deviation rate, H is the number of ore particles at each level detected by various segmentation algorithms, and E is the number of ore particles at each level obtained through manual screening. A smaller value of ε indicates a higher level of detection accuracy.

### 4.2. Result Analysis and Comparison

First, ore particles of different shapes and sizes were placed on the crawler belt of the collection device. After that, the lens aperture was set to the maximum light intake and the focal length was adjusted to 1 m. When the system was working, the camera connection image parameter settings were completed, and the images were saved in the specified folder. The exposure time, automatic gain, and brightness enhancement were set; the button was pushed to start collecting images; the mode was switched to the continuous image capture mode; and the button to start continuous snapshots was pushed. The industrial camera that comes with the device was used to collect images of the ore particles at an interval of 0.1 s. The parameter settings for the experiments were as follows: number of clusters: 3, λ = 7, population size: 30, number of iterations: 100. The parameters for the KM-FA algorithm were consistent with those described in the original paper. There were actually 716 particles in sample a. Among them, there were 163 particles with a particle size of r ≤ 1, 329 particles with a particle size of 1 < r ≤ 5, 164 particles with a particle size of 5 < r ≤ 10, 38 particles with a particle size of 10 < r ≤ 15, and 22 particles with a particle size of r > 15.

**Figure 11:**
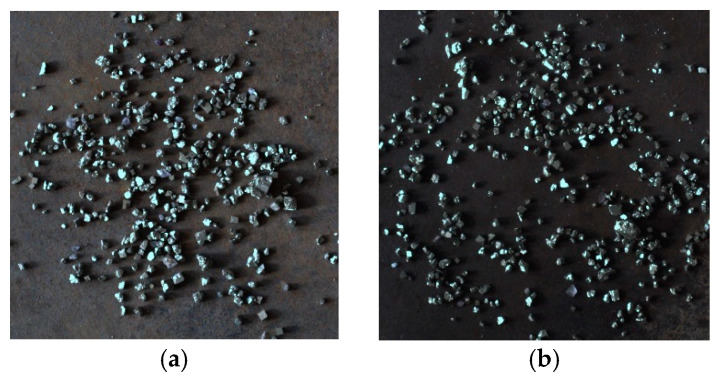
Two kinds of images of ore particle samples. (**a**) Good lighting; (**b**) poor lighting.

A total of 400 images were collected, among which 200 were working conditions with good lighting conditions, with the samples shown in Figure 11a, and 200 were working conditions with poor lighting conditions, with the samples shown in Figure 11b.

**Figure 12:**
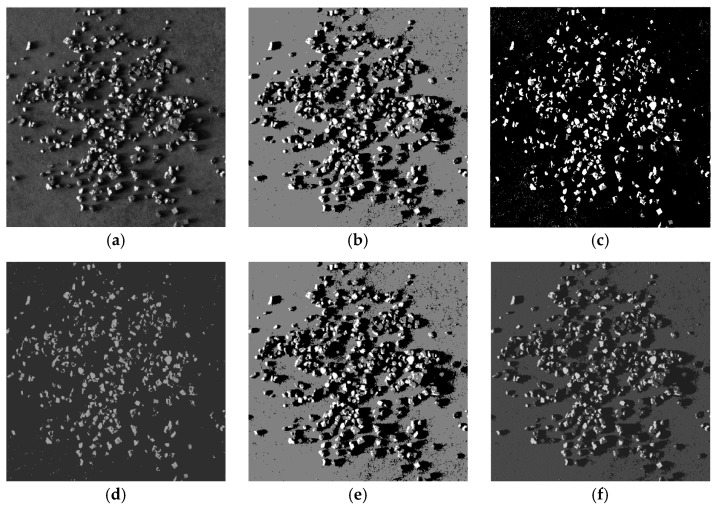
Image segmentation of various algorithms for sample a. (**a**) Original image; (**b**) image of mountain clustering (**c**) image of K-means; (**d**) image of KM-FA; (**e**) image of GK-means; (**f**) image of IGK-means.

**Figure 13:**
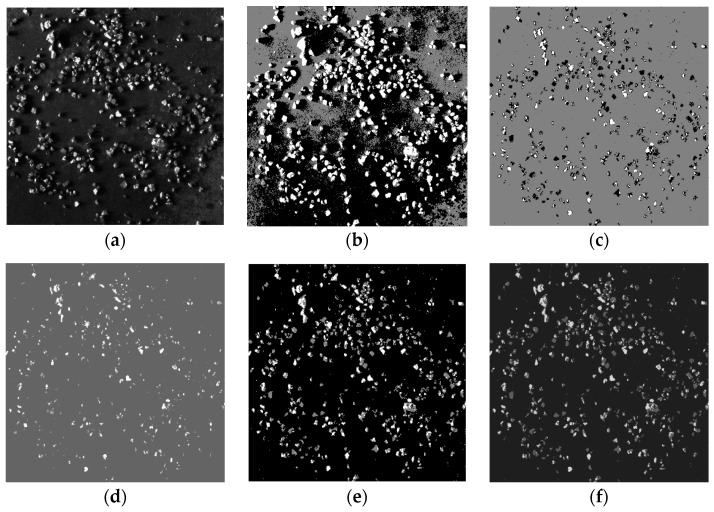
Image segmentation of various algorithms for sample b. (**a**) Original image; (**b**) image of mountain clustering (**c**) image of K-means; (**d**) image of KM-FA; (**e**) image of GK-means; (**f**) image of IGK-means.

**Figure 14:**
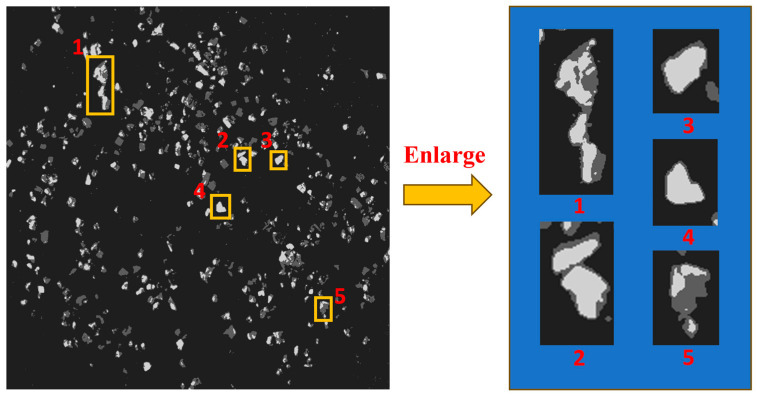
Segmentation details of IGK-means (Numbers 1–5 correspond to enlarged images of some segmentation details).

As shown in Figure 12 and Figure 13, the algorithm proposed in this paper demonstrated significantly better detail handling and overall performance in the ore particle image segmentation experiments compared to the other four algorithms. It achieved higher segmentation accuracy and superior quality. Figure 14 is an enlarged image of some segmentation details of Figure 13f. The experimental results summarizing the segmentation performance of each algorithm are presented in Table 1 and Table 2.

From Table 1, it can be seen that under well-lit conditions, the proposed algorithm achieved the highest PSNR of 23.95 dB, an improvement of 10.06 dB compared to the original algorithm, and it is increased by approximately 72.43%. In addition, the proposed algorithm achieved the highest FSIM of 0.2733, reflecting an enhancement of approximately 48.69%. From Table 2, it can be observed that under poor lighting conditions, both the K-means algorithm and the KM-FA algorithm had significantly reduced segmentation quality, but the algorithm proposed in this paper still achieved a high level of segmentation, with both PSNR and FSIM being the highest. These results indicate that the proposed method effectively addressed the problem of getting stuck in local optima and significantly improved the segmentation quality of the ore particle images. Overall, the results demonstrate that the algorithm presented in this paper not only excels in terms of accuracy under good lighting conditions but also maintains a high level of performance in challenging low-light environments. This highlights its strong adaptability and potential for practical applications, where lighting conditions can often be unpredictable. The runtime compared in this paper refers to the time measured from the moment the cluster centers were output until the image segmentation was completed. The results show that the improved algorithm proposed in this paper reduced the runtime compared to the original algorithm. Moreover, compared to the GK-means, the proposed algorithm significantly improved segmentation quality within a similar runtime, demonstrating its high segmentation efficiency.

**Figure 15:**
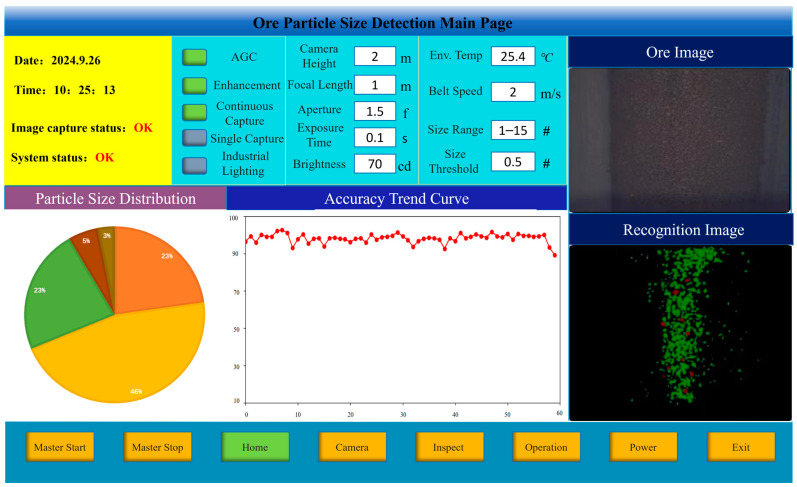
The startup interface of the ore particle detection system.

In order to verify the impact of the ore particle images segmented by the proposed algorithm on ore particle size detection, the ore particle size was artificially divided into seven intervals: r ≤ 1, 1 < r ≤ 5, 5 < r ≤ 10, 10 < r ≤ 15, and r > 15 (where r is the particle size of the ore, in mm). By comparing the segmentation results of the proposed algorithm with the results of manual sieving, the accuracy of the method was verified. The startup interface of the system software is shown in Figure 15.

**Figure 16:**
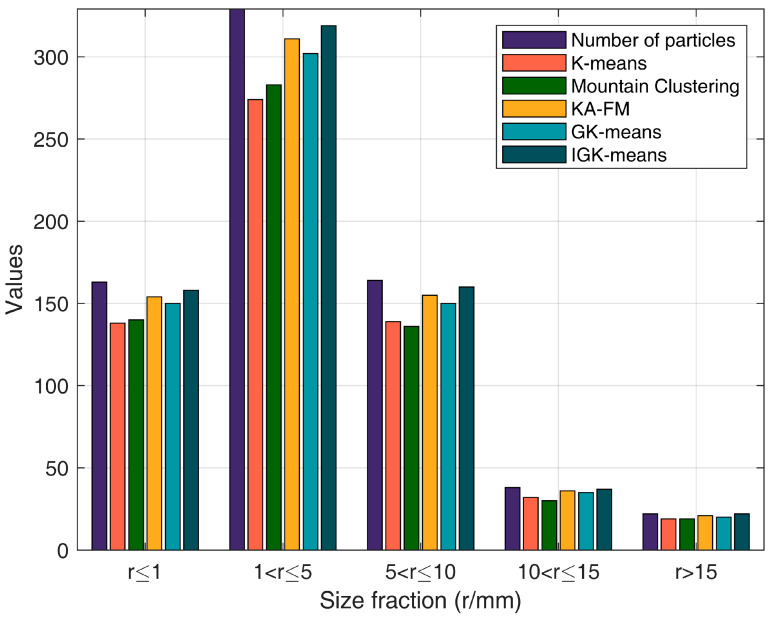
Distribution diagrams of particle size detection for each algorithm.

Based on the ore particle segmentation image, a connected component analysis was first performed to determine the number of connected components and the area of each component. Subsequently, the pixel count within each component was converted into the actual ore area. The horizontal projection of the ore was approximated as a circle, and the ore particle size r was then estimated using the area formula for a circle, S = πr^2^. Given that the industrial camera’s height, focal length, and resolution were fixed, it was found that a 5 mm ore particle corresponded to an average of 55 pixels, while a 10 mm particle corresponded to 201 pixels. Consequently, the area of the connected components in the image, ranging from 36 to 108 pixels, was considered to correspond to ore particle sizes of between 5 mm and 10 mm. Similar mappings can be applied to other pixel area ranges to determine the corresponding ore particle sizes. The results of the ore particle size detection are presented in Figure 16. The deviation rates of particle size detection using the segmented images by each algorithm are shown in Table 3.

It can be seen from Table 3 that the deviation rate of particle size detection by using the K-means to segment images was the highest, while the deviation rate of the method proposed in this paper was the smallest and its detection accuracy was the highest. Moreover, the deviation rate was 0% when r > 15. This indicates that the image segmentation method put forward in this paper can be applied well to the particle size detection of ore particles, improving the efficiency and accuracy of detection and proving the effectiveness and superiority of the algorithm in this paper.

In actual industrial production, according to national standards and preliminary research, the requirement for the detection error rate is that the deviation rate be less than 5% and the segmentation time be less than 0.5 s; the faster, the better. Generally, the pixel accuracy (PA) should reach more than 90%. For some scenarios with extremely high precision requirements, such as the inspection of aerospace components and the production of high-end optical instruments, the PA may need to reach more than 95% or even 98% to ensure the quality and safety of products. For high-quality image segmentation, the intersection over union (IoU) needs to reach 0.6 or even 0.7 or above to ensure the accurate segmentation of the target object. The above-mentioned experiments have proven that the images processed by the algorithm in this paper all meet the requirements of industrial production.

**Figure 17:**
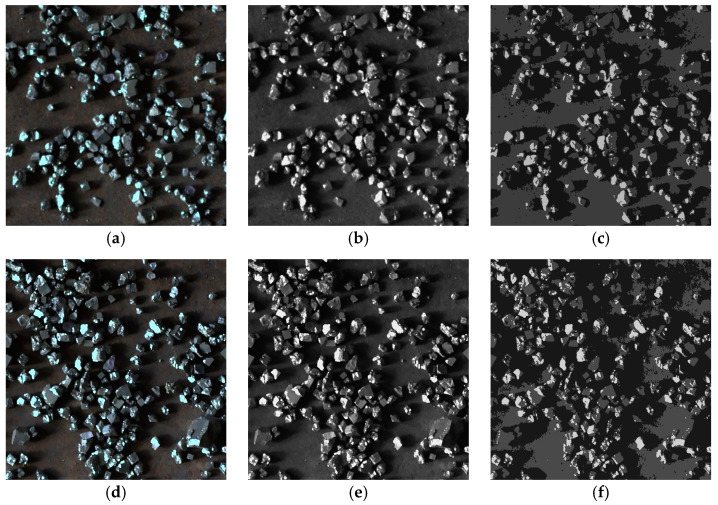
Segmentation of ore images with different degrees of adhesion. (**a**) Sample with general adhesion; (**b**) original image with general adhesion; (**c**) image of IGK-means; (**d**) sample with severe adhesion; (**e**) original image with severe adhesion; (**f**) image of IGK-means.

In order to discuss the performance of the algorithm in this paper when dealing with ore particles with different degrees of adhesion, 400 images were collected. Among them, 200 images were of general adhesion, with the sample shown in Figure 17a, and 200 images were of severely adhered images, with the sample shown in Figure 17d. In this paper, the contour solidity [47] of ore particles was taken as the basis for distinguishing the degree of adhesion of ore particles. The higher the contour solidity, the higher the degree of adhesion. The sample image was collected at the industrial site, and the original image was obtained by performing grayscale processing on the sample image. As can be seen from Figure 17, the algorithm in this paper still had a high degree of precision when handling dense and adhered particles.

For sample a, the intersection over union (IoU) was 0.76173 and the pixel accuracy (PA) was 97.84%; for sample b, the IoU was 0.72434 and the PA was 97.25%. The experiments demonstrated that when IGK-means dealt with ore particles of varying degrees of adhesion and higher density, it still maintained good accuracy and processing capabilities, meeting the requirements of the preliminary research.

### 4.3. Parameter Discussion

In order to discuss the impact of the value of λ in Formula 19 on the performance of the algorithm in this paper, with the other parameters remaining unchanged, image segmentation and particle size detection experiments on ore particle sample a were carried out for λ ranging from 1 to 10. The most suitable value of λ was found by comparing the image segmentation performance indicators (PSNR and FSIM) and the deviation rate of particle size detection. Among them, the results of the image segmentation experiment are shown in Table 4, and the results of the deviation rate of particle size detection are shown in Table 5.

As observed from Table 4 and Table 5, when the value of λ was within the range of [4, 7], the image segmentation performance was relatively high and the deviation rate of particle size detection was relatively low. Therefore, it is recommended that the value of a be within the range of [4, 7]. When λ = 7, the PSNR and FSIM values of the segmented image achieved their respective maxima, indicating that the algorithm’s segmentation accuracy was optimal under this condition. Concurrently, the deviation rate of particle size detection across all particle size grades was minimized, signifying the highest accuracy in particle size detection. Therefore, based on this analysis, the optimal value of λ for this paper was determined to be 7.

## 5. Conclusions

To enhance the segmentation quality of ore particle images, this study proposes an innovative approach combining the K-means algorithm with a hybrid optimization strategy, implemented through the analysis of acoustic signal properties. The proposed method improves the classical grey wolf optimization (GWO) algorithm by refining its convergence factor mechanism and incorporating the migration and spiral attack strategies of the seagull algorithm to enhance late-stage optimization capabilities. Additionally, the intelligent optimization strategy is integrated into the iterative process of K-means to achieve superior segmentation performance. To validate the effectiveness and robustness of the proposed approach, an industrial application experiment was conducted, accompanied by comparative analyses. Experimental results confirm that the proposed method consistently outperforms alternative approaches in segmentation accuracy and quality. In addition, when the method proposed in this paper is applied to particle size detection, the results show that the accuracy of the particle size detection results using the algorithm of this paper is greatly improved, and the detection is more precise.

Despite the promising performance of the IGK-means method in controlled laboratory environments and its demonstrable advantages over existing image segmentation techniques, the approach exhibits several limitations: (1) The IGWO_SOA involves computationally intensive processes in each iteration, including calculating the distance between each “wolf” and the prey (optimal solution), updating positions, and evaluating fitness functions. These repetitive operations result in exponential growth in computational complexity as the data scale and dimensionality increase, significantly impacting runtime efficiency. (2) The image acquisition experiments primarily simulated ore conditions on a conveyor belt, which may not fully capture the complexities of real-world ore particle collection scenarios, which often involve harsh and variable environmental conditions. Thus, the method’s practical adaptability remains a subject for further investigation. Future research will aim to address these limitations by optimizing the computational structure of the algorithm, improving its runtime efficiency, and enhancing its robustness and adaptability for deployment in more complex and realistic industrial environments.

## Figures and Tables

**Table 1:** Segmentation performance of each algorithm for sample a.

Algorithm	PSNR	FSIM	Runtime	IoU	PA
K-means	13.89 dB	0.1838	0.65283 s	0.081045	33.65%
MC	11.68 dB	0.0865	0.35960 s	0.475740	94.40%
KM-FA	20.05 dB	0.1700	0.31806 s	0.629950	96.68%
GK-means	14.02 dB	0.1959	0.09314 s	0.083367	35.67%
IGK-means	23.95 dB	0.2733	0.09909 s	0.787510	98.64%

**Table 2:** Segmentation performance of each algorithm for sample b.

Algorithm	PSNR	FSIM	Runtime	IoU	PA
K-means	8.32 dB	0.0894	0.62799 s	0.039372	8.91%
MC	8.57 dB	0.0361	0.28812 s	0.050801	56.65%
KM-FA	11.32 dB	0.0932	0.35398 s	0.593970	97.08%
GK-means	11.80 dB	0.0938	0.09818 s	0.096261	85.88%
IGK-means	24.24 dB	0.2607	0.09364 s	0.727900	98.09%

**Table 3:** The deviation rate of particle size detection for sample a.

Algorithm	*ε*_r≤1_	*ε*_1<r≤5_	*ε*_5<r≤10_	*ε*_10<r≤15_	*ε*_r>15_
K-means	15.33742%	16.71733%	15.24390%	15.78947%	13.63636%
MC	14.11043%	13.98176%	17.07317%	21.05263%	13.63636%
KA-FM	5.52147%	5.47113%	5.48781%	5.26316%	4.52546%
GK-means	7.97546%	8.51064%	8.53659%	7.89474%	9.09090%
IGK-means	3.06749%	3.03851%	2.43902%	2.63158%	0%

**Table 4:** Image segmentation performance with different values of λ.

	λ = 1	λ = 2	λ = 3	λ = 4	λ = 5	λ = 6	λ = 7	λ = 8	λ = 9	λ = 10
PSNR/dB	23.93	23.90	23.82	23.94	23.95	23.95	23.95	23.94	23.95	23.90
FISM	0.2723	0.2817	0.2735	0.2817	0.2732	0.2645	0.2733	0.2644	0.2724	0.2734

**Table 5:** The deviation rate of particle size detection with different values of λ.

	*ε*_r≤1_	*ε*_1<r≤5_	*ε*_5<r≤10_	*ε*_10<r≤15_	*ε*_r>15_
λ = 1	4.29448%	3.64742%	4.26829%	2.63158%	0%
λ = 2	4.29448%	4.07524%	4.26829%	2.63158%	0%
λ = 3	4.90798%	4.25532%	4.87805%	2.63158%	0%
λ = 4	3.68098%	3.64742%	3.65854%	2.63158%	0%
λ = 5	3.68098%	3.33435%	3.04878%	2.63158%	0%
λ = 6	3.68098%	3.33435%	3.04878%	2.63158%	0%
λ = 7	3.06749%	3.03851%	2.43902%	2.63158%	0%
λ = 8	4.29448%	3.64742%	3.65854%	2.63158%	0%
λ = 9	3.68098%	3.64742%	3.65854%	2.63158%	0%
λ = 10	4.90798%	4.25532%	4.87805%	2.63158%	0%

**Figure A1:**
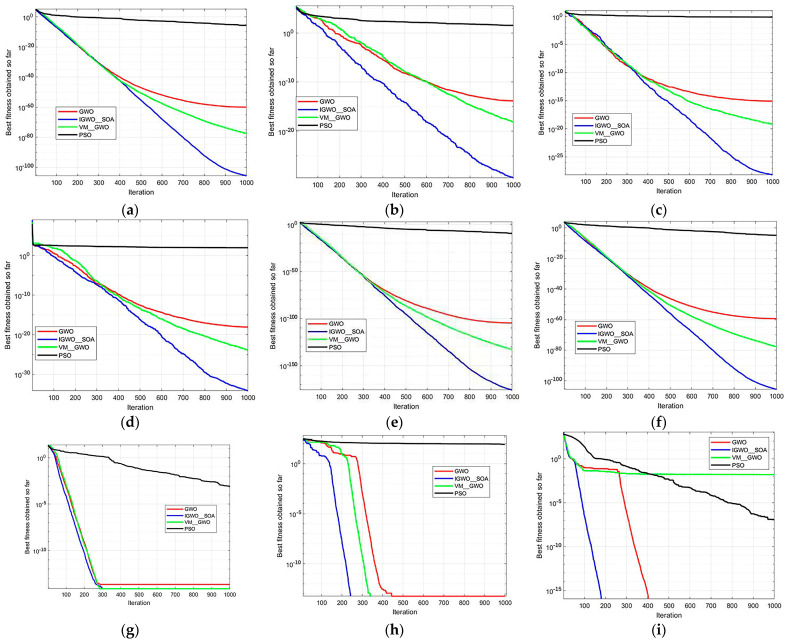
Convergence curves of the four algorithms. (**a**) Sphere; (**b**) Schwefel1.2; (**c**) Schwefel 2.21; (**d**) Zakharov; (**e**) Quartic; (**f**) sum squares; (**g**) Ackley; (**h**) Rastrigin; (**i**) Griewank.

**Figure A2:**
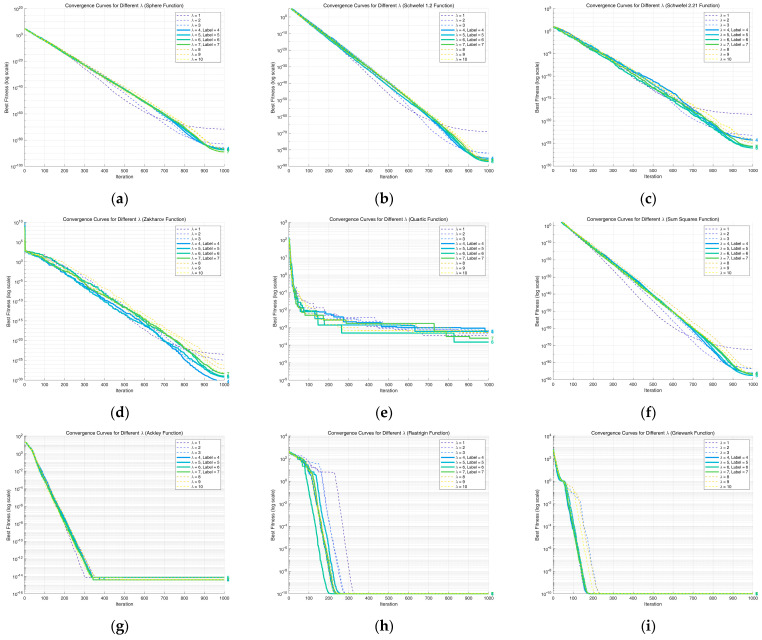
Convergence curves for different λ. (**a**) Sphere; (**b**) Schwefel1.2; (**c**) Schwefel 2.21; (**d**) Zakharov; (**e**) Quartic; (**f**) sum squares; (**g**) Ackley; (**h**) Rastrigin; (**i**) Griewank.

## Data Availability

Data are contained within the article.

## Appendix A. IGWO_SOA Simulation Analysis

Nine benchmark functions were used to simulate and compare IGWO_SOA, GWO, particle swarm optimization (PSO), and the adaptive weight-adjusted VW_GWO proposed in [11] to verify the superiority of the proposed IGWO_SOA, where $f_{1}$–$f_{6}$ are single-peak functions and $f_{7}$–$f_{9}$ are multi-peak functions. All algorithms were run with a population size of 30 and a maximum iteration number of 1000. In the PSO, the maximum speed of particles was set to 6, $c_{1}=c_{2}=1.5$, and w=0.8. The algorithm was run independently 30 times, and the average value and standard deviation were obtained to reflect the convergence precision of the algorithm under a given iteration number, the optimal value to reflect the search effectiveness of the algorithm, and the variance to reflect the stability of the algorithm. The benchmark functions are shown in Table A1 and Table A2 displays the comparison results, while Figure A1 illustrates the convergence curves. In terms of optimal fitness values, the IGWO_SOA algorithm performed better than the compared algorithms across all nine benchmark functions.

**Table A1:** Test function.

	Function Name	Function Expression	Dimension	Interval	Optimal Value
$f_{1}$	Sphere	$f_{1}\left(x\right)=\overset{n}{\underset{i=1}{\sum }}x_{i}^{2}$	30	[−100, 100]	0
$f_{2}$	Schwefel1.2	$f_{2}\left(x\right)=\overset{n}{\underset{i=1}{\sum }}{\left(\overset{i}{\underset{j-1}{\sum }}x_{j}\right)}^{2}$	30	[−100, 100]	0
$f_{3}$	Schwefel 2.21	$f_{3}\left(x\right)=max_{i}\left\{\left|x_{i}\right|,1⩽i⩽n\right\}$	30	[−10, 10]	0
$f_{4}$	Zakharov	$f_{4}\left(x\right)=\overset{n}{\underset{i=1}{\sum }}x_{i}^{2}+{\left(\overset{n}{\underset{i=1}{\sum }}0.5\cdot i\cdot x_{i}\right)}^{2}+{\left(\overset{n}{\underset{i=1}{\sum }}0.5\cdot i\cdot x_{i}\right)}^{4}$	30	[−5, 10]	0
$f_{5}$	Quartic	$f_{5}\left(x\right)=\overset{n}{\underset{i=1}{\sum }}ix_{i}^{4}+random\left[0,1\right)$	30	[−1.28, 1.28]	0
$f_{6}$	Sum squares	$f_{6}\left(x\right)=\overset{n}{\underset{i=1}{\sum }}i\cdot x_{i}^{2}$	30	[−100, 100]	0
$f_{7}$	Ackley	$f_{7}\left(x\right)=-20\exp\left(-0.2\sqrt{\frac{1}{n}\overset{n}{\underset{i=1}{\sum }}x_{i}^{2}}\right)-\exp\left(\frac{1}{n}\overset{n}{\underset{i=1}{\sum }}\cos\left(2\pi x_{i}\right)\right)+20+e$	30	[−32, 32]	0
$f_{8}$	Rastrigin	$f_{8}\left(x\right)=\left[x_{i}^{2}-10\cos\left(2\pi x_{i}\right)+10\right]$	30	[−5.12, 5.12]	0
$f_{9}$	Griewank	$f_{9}=\frac{1}{4000}\overset{n}{\underset{i=1}{\sum }}{x_{i}}^{2}-\overset{n}{\underset{i=1}{\prod }}\cos\left(\frac{x_{i}}{\sqrt{i}}\right)+1$	30	[−600, 600]	0

**Table A2:** Comparison of test results.

	PSO			GWO			IGWO_SOA			VW_GWO		
	Average	Mean	Variance	Average	Mean	Variance	Average	Mean	Variance	Average	Mean	Variance
$f_{1}$	4.1742 × 10^−6^	2.2645 × 10^−7^	2.8536 × 10^−11^	4.9474 × 10^−58^	3.9822 × 10^−62^	5.2488 × 10^−114^	6.9004 × 10^−104^	6.6648 × 10^−108^	3.6915 × 10^−206^	9.8470 × 10^−77^	2.3703 × 10^−80^	1.9929 × 10^−76^
$f_{2}$	3.1987 × 10^1^	9.5290 × 10^0^	1.9508 × 10^2^	3.2340 × 10^−14^	1.2350 × 10^−20^	2.3496 × 10^−26^	3.9348 × 10^−24^	2.1752 × 10^−31^	2.1406 × 10^−46^	5.0501 × 10^−17^	2.6654 × 10^−25^	2.7364 × 10^−16^
$f_{3}$	1.0148 × 10^0^	6.9649 × 10^−2^	5.1419 × 10^−1^	5.4561 × 10^−14^	2.8531 × 10^−16^	2.2134 × 10^−26^	7.8288 × 10^−28^	3.6628 × 10^−31^	2.7307 × 10^−54^	1.0275 × 10^−19^	2.8586 × 10^−21^	1.8050 × 10^−19^
$f_{4}$	7.3050 × 10^1^	2.7617 × 10^1^	8.7247 × 10^2^	2.2653 × 10^−19^	3.1277 × 10^−22^	3.9730 × 10^−37^	5.3107 × 10^−31^	7.4158 × 10^−38^	4.0787 × 10^−60^	1.1626 × 10^−77^	2.5963 × 10^−77^	4.4216 × 10^−81^
$f_{5}$	2.3858 × 10^0^	3.5743 × 10^−2^	1.4251 × 10^1^	9.1485 × 10^−4^	2.8499 × 10^−4^	3.1087 × 10^−7^	6.2781 × 10^−4^	9.2757 × 10^−5^	1.7933 × 10^−7^	2.0732 × 10^0^	−1.7542 × 10^0^	1.4744 × 10^1^
$f_{6}$	2.6667 × 10^1^	2.0000 × 10^−6^	6.8506 × 10^3^	1.068 × 10^4^	1.1637 × 10^−63^	1.4639 × 10^−117^	2.3028 × 10^−104^	1.3776 × 10^−108^	5.6396 × 10^−207^	1.2169 × 10^−77^	8.0540 × 10^−81^	3.0934 × 10^−77^
$f_{7}$	4.2930 × 10^−2^	2.7226 × 10^−3^	1.0212 × 10^−3^	1.5602 × 10^−14^	1.1102 × 10^−14^	5.8611 × 10^−30^	8.1416 × 10^−15^	7.5495 × 10^−15^	2.6839 × 10^−30^	7.4311 × 10^−15^	3.9968 × 10^−15^	6.49 × 10^−16^
$f_{8}$	6.58 × 10^1^	2.7927 × 10^1^	6.2789 × 10^2^	5.7748 × 10^−1^	0	2.7916	0	0	0	3.3592 × 10^−1^	0	1.4855 × 10^0^
$f_{9}$	1.3513 × 10^−2^	0	2.940 × 10^−4^	3.9765 × 10^−3^	0	6.3058 × 10^−5^	0	0	0	9.2489 × 10^−4^	0	3.5751 × 10^−3^

As seen in Figure A1, the IGWO_SOA algorithm demonstrated rapid convergence to the global optimal value, with faster convergence speed and higher accuracy. In contrast, the convergence speeds of the GWO and PSO algorithms were slower. Compared with the other three algorithms, the optimization results of the IGWO_SOA algorithm are more accurate, with a smaller variance and high algorithm stability. Among them, for the $f_{8}$ and $f_{9}$ test functions, the algorithm converged to the theoretical optimal value of 0. This demonstrates the exceptional adaptability and outstanding efficacy of the IGWO_SOA algorithm in addressing intricate optimization challenges, offering a robust computational approach for tackling real-world engineering problems.

## Appendix B. Analysis of λ Adaptability

In order to ensure the adaptability of λ, nine benchmark functions (as shown in Table A1) were used for testing, and the relevant parameters were kept consistent with those in Appendix A. As can be seen from Figure A2, when λ ranges from 1 to 10, the convergence curves and the optimal values of the algorithm are presented.

As is evident from Figure A2, when the parameter λ assumes values within the range of 4 to 7, the experimental evaluations of the nine benchmark functions demonstrate relatively superior performance. In this specific range, the algorithm not only accelerates its convergence rate significantly but also yields optimal solutions of higher precision and quality, outperforming scenarios where λ takes other values. This experimental outcome aligns closely with the value of a determined through prior experimental investigations.
